# EIF4A3‐Mediated Biogenesis of CircFADS1 Promotes the Progression of Hepatocellular Carcinoma via Wnt/β‐Catenin Pathway

**DOI:** 10.1002/advs.202411869

**Published:** 2025-02-18

**Authors:** Lei Tang, Yang Ji, Chuangye Ni, Zhenggang Xu, Yanjun Shen, Hao Lu, Chuanyong Zhang, Shikun Yang

**Affiliations:** ^1^ Hepatobiliary Center The First Affiliated Hospital of Nanjing Medical University Key Laboratory of Liver Transplantation Chinese Academy of Medical Sciences NHC Key Laboratory of Living Donor Liver Transplantation (Nanjing Medical University) No. 300 Guangzhou Road Nanjing Jiangsu 210029 China; ^2^ Medical College Yangzhou University Yangzhou Jiangsu 225009 China

**Keywords:** circFADS1, GSK3β, HCC, proliferation, Wnt/β‐catenin pathway

## Abstract

Mounting research indicates that circRNAs are pivotal elements in tumorigenesis and progression. Understanding the mechanisms by which circRNAs function in tumors is crucial for identifying undiscovered diagnostic and treatment targets. This research centers on unraveling the mechanisms by which the novel circRNA, circFADS1, influences hepatocellular carcinoma (HCC) progression. CircFADS1 shows elevated expression in HCC and is linked to unfavorable prognosis. Functionally, circFADS1 overexpression accelerates HCC progression through inducing HCC proliferation and inhibited apoptosis. Mechanistically, RNA‐seq analysis demonstrates its connection to the Wnt/β‐catenin pathway. Moreover, circFADS1 interacts with GSK3β and promotes its ubiquitination and degradation by recruiting the ubiquitin ligase RNF114 while EIF4A3 facilitates the biogenesis of circFADS1. Additionally, circFADS1 is closely linked to lenvatinib resistance. Overall, this study reveals that circFADS1 regulates GSK3β function, influencing the progression of hepatocellular carcinoma. The EIF4A3/circFADS1/GSK3β/β‐catenin axis is discovered to hold promise as a novel therapeutic target for hepatocellular carcinoma, while circFADS1 is also a significant factor in lenvatinib resistance.

## Graphical abstract

In this study, a novel circRNA, circFADS1 is identified, which is expressed at higher levels in HCC. circFADS1 is associated with poor prognosis in HCC patients and promotes HCC cell proliferation while inhibiting apoptosis. Mechanistically, circFADS1 exerts its biological function through the EIF4A3/GSK3β/β‐catenin axis and is closely associated with resistance to lenvatinib.

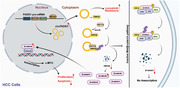

## 1 Introduction

Hepatocellular carcinoma (HCC) comprised ≈75–85% of primary liver cancers, ranked sixth in global prevalence and third in cancer‐related mortality.^[^ ^1^ ^]^ Given the late presentation of symptoms, more than half of the patients are already in the advanced stage, where surgical intervention alone proves insufficient for effective treatment.^[^ ^2^, ^3^ ^]^ Sorafenib, the first systemic therapy approved for advanced HCC, has significantly improved overall survival (OS) in these patients. Due to its non‐inferiority to sorafenib, lenvatinib was authorized as the second first‐line targeted therapy.^[^ ^4^, ^5^ ^]^ However, not all HCC patients benefit from targeted therapy, and resistance to lenvatinib is progressively emerging as a significant challenge in its application.^[^ ^4^ ^]^ Thus, it is crucial to uncover new molecular drivers of HCC and therapeutic targets, and pinpoint targets that can overcome lenvatinib resistance.

CircRNAs represent a category of non‐coding RNAs generated through backsplicing. Due to their lack of 5′ caps and 3′ poly(A) tails, circRNAs exhibit greater stability compared to linear RNAs.^[^ ^6^ ^]^ The stability conferred by their circular structure holds potential for therapeutic applications.^[^ ^7^ ^]^ Given their role in regulating various cellular activities and pathological processes, the dysregulated expression of circRNAs is often linked to tumorigenesis and clinical prognosis.^[^ ^8^, ^9^ ^]^ A substantial body of research indicates that circRNAs have three primary functions: functioning as miRNA sponges, engaging in protein interactions, and encoding peptides. As an illustration, hsa_circ_0119412 enhances ZBED3 expression by serving as a sponge of miR‐1298‐5p, which eventually promotes gastric cancer tumorigenesis.^[^ ^10^ ^]^ Chen et al. discovered that circACTN4 can interact with FUBP1 to enhance MYC expression, thereby promoting the initiation and progression of breast cancer.^[^ ^11^ ^]^ Zhang et al. identified that circMET encodes MET404, which drives glioblastoma tumorigenesis.^[^ ^12^ ^]^ Furthermore, circPAK1, a driver of HCC, is implicated in the lenvatinib resistance.^[^ ^13^ ^]^ Our previous research identified circERBIN, which functions as a ceRNA for miR‐1263, targeting cyclin‐dependent kinase 6 and regulating the G1/S transition.^[^ ^14^ ^]^ Nevertheless, a significant portion of circRNAs remains unidentified and uninvestigated.

In the present study, we pinpointed circFADS1, a newly discovered circRNA, as the central focus of our investigation by mining Gene Expression Omnibus (GEO) databases. The data indicated that circFADS1 was markedly overexpressed in HCC, correlating with reduced patient survival and worsened prognosis, while in vivo and in vitro studies confirmed its role in driving HCC progression. Mechanistically, EIF4A3 promotes the biogenesis of circFADS1, which, as revealed by high‐throughput sequencing, activates the Wnt/β‐catenin pathway. RNA pulldown assays combined with mass spectrometry revealed that circFADS1 directly binds to GSK3β, enhancing its ubiquitination and eventual degradation. Additional investigations revealed that circFADS1 recruits the E3 ubiquitin ligase RNF114, thereby enhancing its interaction with GSK3β. Moreover, in the lenvatinib‐resistant cell lines we developed, circFADS1 expression was significantly elevated compared to the parental cell lines, while knockdown of circFADS1 enhanced their sensitivity to lenvatinib. Taken together, circFADS1 holds promise as an innovative treatment target for HCC and a critical target for overcoming lenvatinib resistance.

## 2 Results

### 2.1 CircFADS1 Recognition and Characterization in HCC

**Figure 1:**
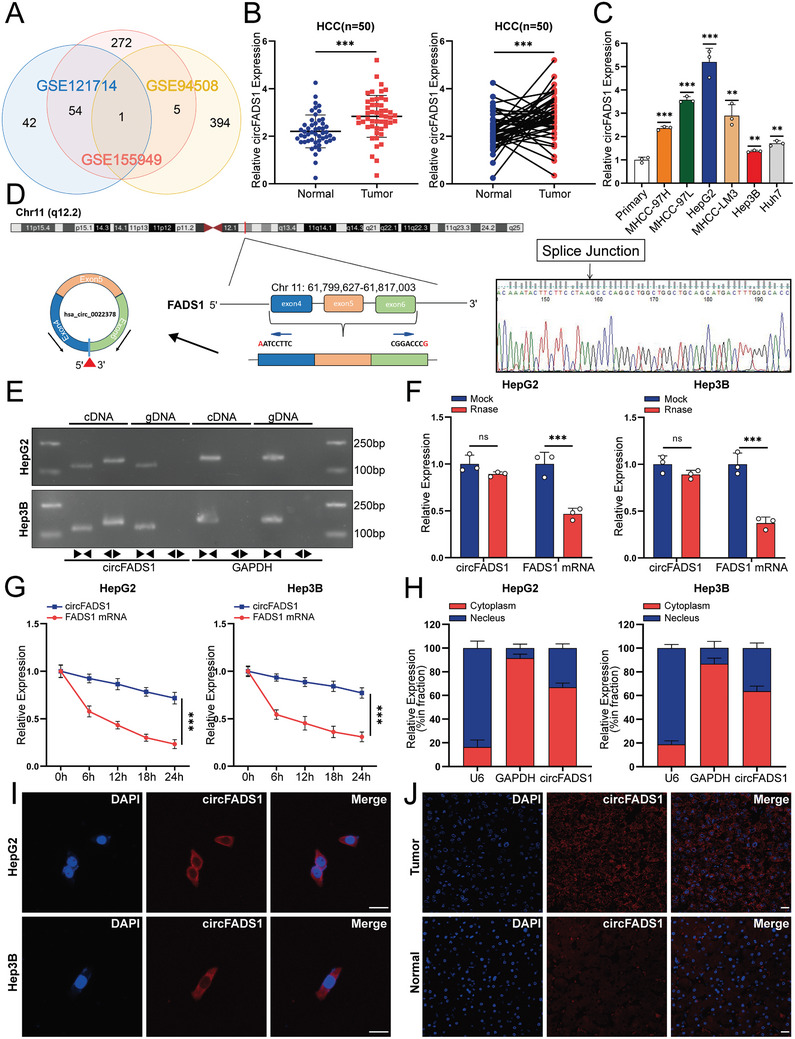
Identification and characteristics of circFADS1. A) A Venn diagram illustrating the intersection of upregulated circRNAs identified from the analysis of GSE121714, GSE155949, and GSE94508 datasets, with only one circRNA, has‐circ‐0022378(circFADS1), was identified. B) The expression of circFADS1 in the 50 paired HCC and adjacent tissues by qRT‐PCR. C) qRT‐PCR was used to analyze circFADS1 expression levels across various cultured HCC cell lines (MHCC‐97H, MHCC‐97L, HepG2, MHCC‐LM3, Hep3B, and Huh7) as well as in primary hepatocytes. D) An annotated genomic region of circFADS1 was illustrated, and Sanger sequencing was performed to verify the back splicing of circFADS1. E) PCR and agarose gel electrophoresis analysis were performed to detect the presence of circFADS1 and FADS1 in cDNA and gDNA samples from HCC cells using divergent and convergent primers. F) After RNase R treatment, the expression levels of circFADS1 and linear FADS1 were measured in HepG2 and Hep3B cells. G) The expression levels of circFADS1 and linear FADS1 were measured in HepG2 and Hep3B cells after actinomycin D treatment. H) Results from the nuclear‐cytoplasmic fractionation assay revealed that circFADS1 predominantly resides in the cytoplasm of HCC cells, with GAPDH and U6 serving as controls for cytoplasmic and nuclear localization, respectively. I,J) FISH analysis revealed that circFADS1 was mostly distributed in the cytoplasm. Scale bar: 20 µm., Scale bar: 50 µm. ^*^ *p* < 0.05; ^**^ *p* < 0.01; ^***^ *p* < 0.001. Data were shown as mean ± SEM.

To identify circRNAs integral to HCC advancement, we utilized the GEO database in this study. GSE121714, GSE155949, and GSE94508 were used to identify highly expressed circRNAs in HCC. The results showed that only circFADS1 (has_circ_0022378) was highly expressed across all these three GSE datasets (logFC > 0.5, adj.P.Val < 0.1) (**Figure** **1**A).

Thus, we conducted qRT‐PCR on 50 pairs of HCC and adjacent non‐tumor tissues, revealing that circFADS1 is significantly overexpressed in tumors compared to normal liver tissues (Figure 1B). Subsequently, HCC patients were ranked by qRT‐PCR results and categorized into high (*n* = 25) and low (*n* = 25) circFADS1 expression groups. As illustrated in **Table** **1**, increased circFADS1 expression is strongly correlated with larger tumor size (*p* = 0.0227), higher TNM classification (*p* = 0.0107), and poorer Edmondson grade (*p* = 0.0235). Moreover, patients exhibiting high circFADS1 expression had shorter OS (Figure S1A, Supporting Information). Across six HCC cell lines, with human primary hepatocytes as the control, HepG2 cells exhibited the highest circFADS1 expression levels, while Hep3B cells had the lowest (Figure 1C), which were used for subsequent studies.

**Table 1:** Correlation between circFADS1 expression and clinicopathological features in HCC tissues (*n* = 50, χ2 ‐test).

	circFADS1 Expression		
Variables	high	low	*P*‐value
	25	25	
**Age (year)**			0.5710
<60	14	12	
≥60	11	13	
**Gender**			0.7760
Male	13	14	
Female	12	11	
**Liver cirrhosis**			0.3821
Yes	17	14	
No	8	11	
**AFP (ng mL^−1^)**			0.4795
≤200	6	4	
>200	19	21	
**HBsAg**			0.5558
Positive	17	15	
Negative	8	10	
**Tumor size**			0.0227*
<5 cm	18	10	
≥5 cm	7	15	
**TNM stage**			0.0107*
I–II	9	18	
III–IV	16	7	
**Edmondson grade**			0.0235*
I–II	8	16	
III–IV	17	9	

^*^ *P* < 0.05.

According to circBase, circFADS1 is derived from the FADS1 gene located on chromosome 11 (q12.2).^[^ ^15^ ^]^ The annotation for circFADS1 includes exons 4, 5, and 6. Sanger sequencing confirmed the back‐splicing of circFADS1 (Figure 1D). To further validate the circular form of circFADS1, qRT‐PCR products were analyzed via agarose gel electrophoresis using both divergent and convergent primers, which indicated that circFADS1 was only amplified by divergent primers in cDNA instead of gDNA (Figure 1E). With RNase R and actinomycin D treatment, we confirmed the greater stability of circFADS1 compared to linear FADS1 (Figure 1F,G).

Next, we utilized cytoplasmic‐nuclear separation and FISH, discovering that circFADS1 was predominantly localized in the cytoplasm of HCC cells (Figure 1H–J). These findings indicate that circFADS1 is markedly upregulated in HCC tissues and primarily localized in the cytoplasm of HCC cells.

### 2.2 CircFADS1 Facilitated HCC Cells Proliferation and Inhibited Apoptosis in Vitro

**Figure 2:**
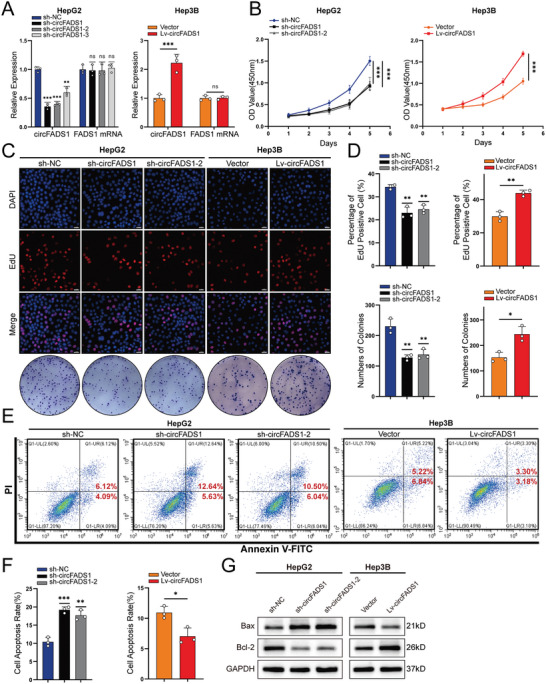
The role of circFADS1 in HCC in vitro. A) Stable circFADS1 knockdown and overexpression HCC cell lines, along with their respective controls, were established, and qRT‐PCR was employed to verify their expression levels of circFADS1 and linear FADS1. B) The proliferation of sh‐circFADS1 and Lv‐circFADS1 cells, compared to their control counterparts, was assessed using the CCK8 assay. C,D) EdU and colony formation assays were performed on HCC cells with circFADS1 knockdown or overexpression. E,F) The percentage of apoptotic cells of sh‐circFADS1 or Lv‐circFADS1 cells was analyzed by Annexin V‐FITC/PI staining assay. G) BAX and Bcl‐2 protein levels in HCC cells were assessed by western blotting. ^*^ *p* < 0.05; ^**^ *p* < 0.01; ^***^ *p* < 0.001. Data were shown as mean ± SEM.

To further investigate how circFADS1 affects the progression of HCC in vitro, we infected HepG2 cells with three different shRNAs specific to the conjunction to establish knockdown cell lines. Meanwhile, Hep3B cells were infected with lentivirus vectors to establish stable cell lines overexpressing circFADS1. Cell lines of the knockdown and overexpression of circFADS1 were both validated by qRT‐PCR (**Figure** **2**A). Due to the higher knockdown efficiency of sh‐circFADS1 and sh‐circFADS1‐2, these two shRNAs were used for further functional experiments on HepG2 cells.

CCK‐8, EdU, and colony formation assays revealed that circFADS1 knockdown significantly attenuated HCC cell proliferation, while circFADS1 overexpression produced opposite effects (Figure 2B–D). Simultaneously, we found that circFADS1 knockdown promoted apoptosis in HepG2 cells and Hep3B cells overexpressing circFADS1 underwent less apoptosis (Figure 2E,F), with western blot results for BAX and Bcl‐2 proteins further validating these findings (Figure 2G). Taken together, these results suggested that circFADS1 enhances proliferation and suppresses apoptosis of HCC cells.

### 2.3 CircFADS1 was Involved in the Wnt/β‐Catenin Pathway

**Figure 3:**
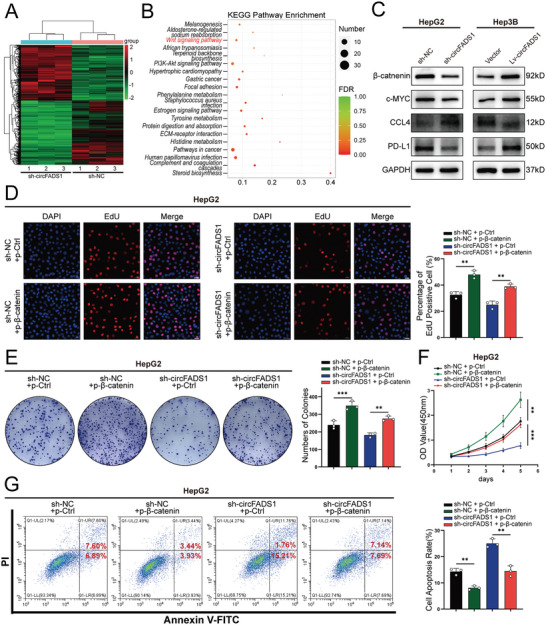
CircFADS1 mediates oncogenic effects on HCC by inactivating the Wnt/β‐catenin pathway. A) Heatmap showed the RNA‐seq result in circFADS1 knockdown and control HepG2 cells. B) Pathway enrichment analysis of differentially expressed genes in RNA‐sequence data. C) The protein level of β‐catenin, c‐MYC, CCL4, and PD‐L1 in HepG2 and Hep3B cells after upregulating or downregulating circFADS1. Rescue experiments of knocking down circFADS1 with overexpressing β‐catenin in HepG2 cells and their controls were conducted, including D) EdU assays (Scale bar, 50 µm), E)colony formation assays, F) CCK‐8 assays and G) apoptosis assessments. ^*^ *p* < 0.05; ^**^ *p* < 0.01; ^***^ *p* < 0.001. Data were shown as mean ± SEM.

To explore the molecular mechanisms of circFADS1 involved in HCC development, RNA‐sequencing was conducted to compare variably expressed RNAs between the sh‐circFADS1 and sh‐NC cohorts in HepG2 cells (**Figure** **3**A). After running the KEGG pathway enrichment, we found that circFADS1 is correlated with the Wnt/β‐catenin pathway (Figure 3B). Building on circFADS1's tumor‐promoting effects and RNA‐seq results, we examined its impact on the Wnt/β‐catenin pathway. As is well known, β‐catenin is the key protein of the Wnt/β‐catenin pathway, while the activation of the pathway leads to the decrease of the phosphorylation of β‐catenin, which results in its accumulation and subsequent translocation into the nucleus to translate genes regulating cell proliferation and survival.^[^ ^16^ ^]^ Therefore, Western blot analysis was performed on Hep3B and HepG2 cell lines to validate the expression levels of β‐catenin and the downstream c‐MYC. The results revealed a decrease in the expression of β‐catenin and c‐MYC in the circFADS1 knockdown group compared to the control group, whereas an opposite trend was observed in the circFADS1 overexpression group (Figure 3C). Meanwhile, studies have shown that the Wnt/β‐catenin pathway is closely associated with anti‐tumor immune responses. Huang et al. discovered that Rh‐1, a novel inhibitor of the Wnt/β‐catenin pathway, can upregulate CCL4 to promote T‐cell infiltration into tumor tissue and synergize with PD‐1 therapy.^[^ ^17^, ^18^ ^]^ Therefore, we explored whether targeting circFADS1 could induce similar immune‐related changes. Additional western blot analysis revealed that the knockdown of circFADS1 increased the expression of CCL4 and decreased the expression of PD‐L1, whereas overexpression of circFADS1 showed the opposite effect (Figure 3C). Taken together, circFADS1 can regulate the Wnt/β‐catenin pathway and has the potential to modulate antitumor immunity.

To further investigate the effects of β‐catenin and circFADS1 on HCC, we respectively transfected HepG2 and Hep3B cells with β‐catenin overexpression plasmid and siRNA, and the transfection efficiency was verified (Figure S1B,C, Supporting Information). The reversal of reduced HCC cell proliferation from circFADS1 knockdown by β‐catenin overexpression was confirmed through EdU, colony formation assays, and CCK‐8 assays, while β‐catenin knockdown reduced the oncogenic effects induced by circFADS1 overexpression (Figure 3D–F; Figure S1D–F, Supporting Information). Next, flow cytometry revealed that β‐catenin overexpression could reverse the increased apoptosis caused by circFADS1 knockdown, while β‐catenin knockdown showed the opposite effect (Figure 3G; Figure S1G, Supporting Information). In summary, circFADS1 drives HCC progression by facilitating the activation of the Wnt/β‐catenin pathway.

### 2.4 CircFADS1 Interacted with GSK3β in HCC Cells

**Figure 4:**
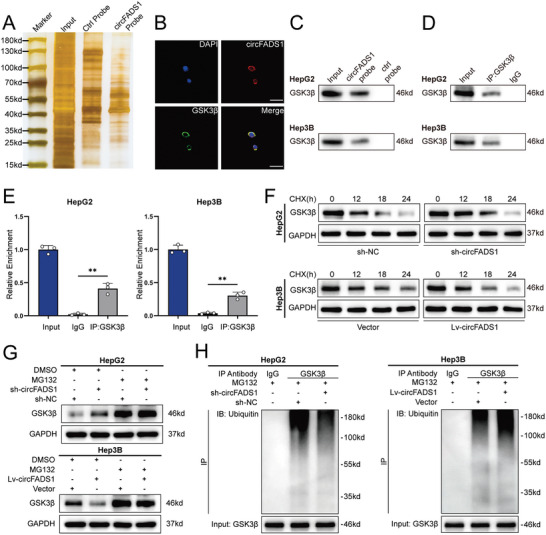
CircFADS1 interacts with GSK3β and promotes its ubiquitination. A) Silver staining was performed on the products obtained from RNA pulldown assays using circFADS1 and control probes. B) RNA FISH and immunofluorescence analysis of the localization of circFADS1 (red) and GSK3β (green). Scale bar, 20 µm. C) Western blotting and RNA pull‐down assays confirmed the presence of GSK3β in the precipitates retrieved with the circFADS1 probe. RIP assay validated the interaction between GSK3β and circFADS1. D) Western blotting was used to assess the IP efficiency of the GSK3β antibody. E) The relative enrichment of circFADS1 in the GSK3β antibody precipitates compared to the input group. F) HepG2 and Hep3B cells with circFADS1 knockdown or overexpression were treated with CHX, and GSK3β expression levels were assessed via western blotting, with GAPDH serving as the control. G) HepG2 and Hep3B cells with circFADS1 knockdown or overexpression were treated with MG132 or DMSO, and GSK3β expression levels were assessed via western blotting, with GAPDH serving as the control. H) Co‐IP and western blotting were used to assess the ubiquitination of GSK3β in HCC cells. ^*^ *p* < 0.05; ^**^ *p* < 0.01; ^***^ *p* < 0.001. Data were shown as mean ± SEM.

Building upon our prior findings, we proceeded to delve deeper into elucidating the potential mechanism underlying the oncogenic function of circFADS1 in hepatocellular carcinoma (HCC). As outlined above, circRNAs possess three primary biological functions: translating peptides, interacting with proteins, and acting as miRNA sponges.^[^ ^19^, ^20^ ^]^ We used the circRNADb database^[^ ^21^ ^]^ to investigate whether circFADS1 has the ability to encode proteins, indicating that the likelihood of circFADS1 containing an opening reading frame (ORF) is minimal. Therefore, we hypothesized that circFADS1 may interact with specific proteins or act as a miRNA sponge to promote HCC. Initially, we conducted an RNA pull‐down assay in HepG2 cells utilizing both the biotin‐labeled probe specific to circFADS1 and a control probe. The precipitates from the RNA pull‐down assay were analyzed using silver staining (**Figure** **4**A). Mass spectrometry (MS) was then used to identify circFADS1‐interacting proteins, identifying 126 unique proteins in the circFADS1 probe precipitates. AGO2, essential for miRNA sponging,^[^ ^22^ ^]^ was not detected in the mass spectrometry results, indicating that circFADS1 may not function as a miRNA sponge. Interestingly, we found that GSK3β, a key component of the Wnt/β‐catenin pathway, was identified in the mass spectrometry results (Figure S2A, Supporting Information).

Previous studies have shown that GSK3β can form a complex with CK1α, APC, and AXIN1, known as the GSK3β‐CK1α‐APC‐AXIN1 complex, which enhances β‐catenin phosphorylation, resulting in its elimination, preventing the translocation into the nucleus and the activation of target gene transcription. To further investigate whether circFADS1 exerts its biological effects through GSK3β, we conducted subsequent experiments. qRT‐PCR confirmed that circFADS1 knockdown or overexpression did not impact GSK3β mRNA levels (Figure S2B, Supporting Information). Furthermore, a Western blot was employed to assess the potential regulatory effect of circFADS1 knockdown or overexpression on the protein expression of GSK3β (Figure S2C, Supporting Information). The results indicated a negative regulation of GSK3β expression on the post‐transcriptional level by circFADS1. Consequently, we hypothesized that circFADS1 may directly interact with GSK3β, leading to the downregulation of GSK3β expression. First, IF indicated colocalization of circFADS1 with GSK3β in the cytoplasm (Figure 4B). The pull‐down assay and western blot confirmed GSK3β in circFADS1 probe precipitates (Figure 4C). The conclusion that GSK3β can bind circFADS1 was derived from the RIP assay using an antibody targeting GSK3β (Figure 4D,E).

Next, we further investigated the mechanism by which circFADS1 reduces the protein expression of GSK3β. Previous studies found that in HCC, GSK3β is induced for ubiquitination‐mediated degradation by MYH9.^[^ ^23^ ^]^ Therefore, we hypothesized that circFADS1 may negatively regulate the expression of GSK3β by promoting its ubiquitination. Building upon this premise, we subjected HCC cells to cycloheximide (CHX) treatment, revealing that circFADS1 knockdown delayed the degradation of GSK3β while overexpression of circFADS1 accelerated the process (Figure 4F). To further confirm that circFADS1 negatively regulates GSK3β expression by promoting its ubiquitination, we transfected HCC cell lines with GSK3β overexpression lentivirus and knockdown siRNA. The transfection efficiency was validated in (Figure S2D,E, Supporting Information). Next, we treated HCC cells with MG132 and the outcomes indicated that the upregulation of GSK3β protein induced by circFADS1 knockdown could be counteracted by MG132. Similarly, the reduction in GSK3β levels resulting from circFADS1 overexpression in Hep3B cells could be reversed by MG132 (Figure 4G). Further analysis was conducted to evaluate the ubiquitination level of GSK3β under different experimental conditions. Co‐immunoprecipitation (Co‐IP) combined with western blotting unveiled that circFADS1 knockdown substantially reduced the ubiquitination level of GSK3β in HepG2 cells, whereas circFADS1 overexpression facilitated the ubiquitination of GSK3β in Hep3B cells (Figure 4H). Taken together, these findings provide substantial evidence that circFADS1 suppresses the level of GSK3β protein through ubiquitination‐mediated degradation in HCC cells.

### 2.5 GSK3β Acts as the Downstream Effector of circFADS1

Building on the discovery that circFADS1 downregulates GSK3β through ubiquitination, we hypothesize that GSK3β serves as the downstream target of circFADS1's biological functions. As shown in (Figures S2F–I and S3A–D, Supporting Information), GSK3β knockdown significantly reversed the impact of circFADS1 downregulation on HepG2 cell growth and apoptosis, while GSK3β overexpression in Hep3B cells counteracted the influence of overexpressing circFADS1. These results confirmed the capacity of circFADS1 to contribute to the progression of HCC through suppressing the level of GSK3β.

### 2.6 GSK3β was Ubiquitinated by RNF114

**Figure 5:**
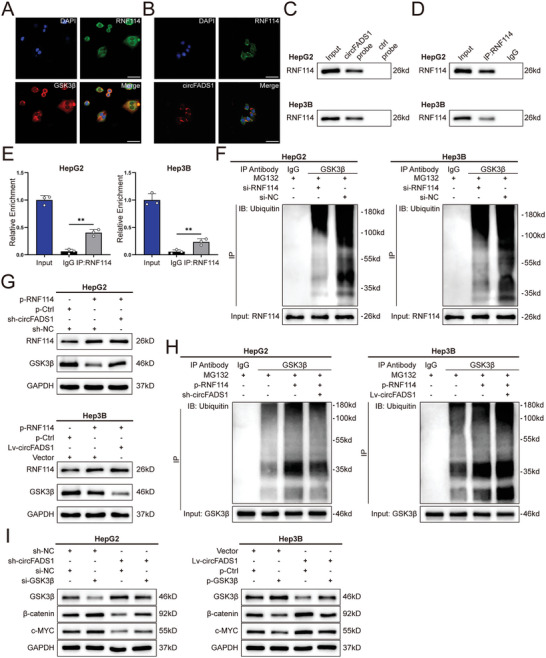
RNF114 acts as the specific E3 ubiquitin ligase for GSK3β, while circFADS1 facilitates their interaction. A) RNA FISH and immunofluorescence analysis of the localization of RNF114 (green) and GSK3β (red). Scale bar, 20 µm. B) RNA FISH and immunofluorescence analysis of the localization of circFADS1 (red) and RNF114 (green). Scale bar, 20 µm. C) Western blotting and RNA pull‐down assays confirmed the presence of RNF114 in the precipitates retrieved with the circFADS1 probe. RIP assay validated the interaction between RNF114 and circFADS1. D) Western blotting was used to assess the IP efficiency of the RNF114 antibody. E) The relative enrichment of circFADS1 in the RNF114 antibody precipitates compared to the input group. F) GSK3β ubiquitination was assessed after RNF114 knockdown. G) Western blotting was used to assess GSK3β protein levels in HCC cells with RNF114 overexpression following circFADS1 knockdown or overexpression. H) Co‐IP and western blotting were used to assess GSK3β ubiquitination in HCC cells following RNF114 overexpression and circFADS1 knockdown or overexpression. I) Western blotting verified the activation of the Wnt/β‐catenin pathway in HCC cells. ^*^ *p* < 0.05; ^**^ *p* < 0.01; ^***^ *p* < 0.001. Data were shown as mean ± SEM.

Furthermore, we explored how circFADS1 mediates GSK3β ubiquitination and degradation in HCC. Plenty of studies have revealed that ubiquitin E3 ligases are key in discerning specific substrates for ubiquitination by binding directly to them.^[^ ^24^ ^]^ We thus hypothesized that, mechanistically, a specific E3 ubiquitin ligase interacts with both circFADS1 and GSK3β, facilitating GSK3β ubiquitination. Interestingly, we identified only one ubiquitin E3 ligase, RNF114, in the mass spectrometry results (Figure S4A, Supporting Information). IF showed that RNF114 is co‐localized with GSK3β and circFADS1 in the cytoplasm (**Figure** **5**A,B). Similarly, RNA pull‐down assays and RIP assays validated the ability of circFADS1 binding with RNF114 (Figure 5C–E). Neither knockdown nor overexpression of circFADS1 affected the protein or mRNA levels of RNF114, indicating that circFADS1 can only recruit and bind to RNF114 without regulating its expression (Figure S4B,C, Supporting Information). We designed an overexpression lentivirus and a knockdown siRNA specific for RNF114 to conduct the following experiment (Figure S4D–G, Supporting Information). We found that knockdown of RNF114 upregulated GSK3β expression while reducing ubiquitination levels (Figure 5F; Figure S4E, Supporting Information). These results indicate that RNF114 is a specific E3 ubiquitin ligase for GSK3β.

### 2.7 CircFADS1 Promotes the Binding of RNF114 to GSK3β

To further investigate the role of circFADS1 between GSK3β and RNF114, we conducted additional studies. Knockdown of circFADS1 can counteract the GSK3β downregulation caused by RNF114 overexpression, while overexpression of circFADS1 further enhances the degradation of GSK3β induced by high levels of RNF114 (Figure 5G). Co‐IP experiments further confirmed that circFADS1 knockdown alleviated the increase in ubiquitination levels caused by RNF114 overexpression. Conversely, overexpression of circFADS1 in Hep3B cells showed the opposite result (Figure 5H). These findings indicate that circFADS1 is crucial for RNF114 binding to GSK3β and its ubiquitination.

Rescue experiments were conducted to additionally confirm whether the downregulation of GSK3β caused by circFADS1 influences the Wnt/β‐catenin pathway, and the results revealed downregulation of Wnt/β‐catenin pathway‐related proteins and their downstream targets induced by circFADS1 knockdown could be reversed by low levels of GSK3β in HepG2 cells. Moreover, overexpression of GSK3β in Hep3B cells counteracted the excessive activation of the pathway induced by high levels of circFADS1 (Figure 5I). In summary, circFADS1 promotes HCC progression through the RNF114/GSK3β/β‐catenin axis.

### 2.8 Biogenesis of circFADS1 is Facilitated by EIF4A3

**Figure 6:**
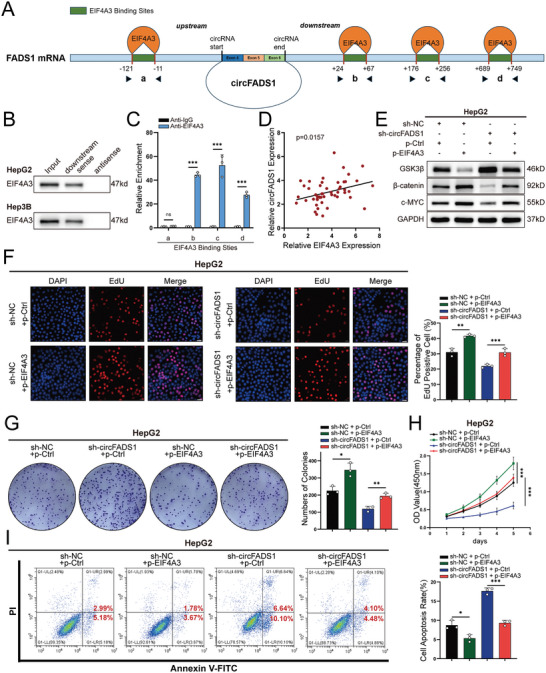
EIF4A3 markedly enhanced the expression of circFADS1. A) Schematic diagram of the EIF4A3 binding sites in the flanking of circFADS1. B) Pull‐down assay with western blot verified the ability of EIF4A3 binding to the downstream of circFADS1 flanking instead of upstream. C) RIP assay to verify EIF4A3 binding at the putative sites. D) The correlation between EIF4A3 and circFADS1 (*n* = 50). E) Western blot of GSK3β, β‐catenin, and c‐MYC under the condition of altered circFADS1 and EIF4A3 expression. Rescue experiments of overexpressing EIF4A3 with knocking down circFADS1 in HepG2 cells and their controls were conducted, including F) EdU assays (Scale bar, 50 µm), G) colony formation assays, H) CCK‐8 assays and I) apoptosis assessments. *p < 0.05; **p < 0.01; ***p < 0.001. Data were shown as mean ± SEM.

Prior research showed that certain RNA‐binding proteins (RBPs) bind to circRNA‐adjacent intronic regions, such as FUS^[^ ^25^ ^]^ and QKI,^[^ ^26^, ^27^ ^]^ contributing to and facilitating circRNA biogenesis. Consequently, we utilized the CircInteractome^[^ ^28^ ^]^ to identify RBPs associated with circFADS1, showing that EIF4A3 has the highest number of binding sites. (**Figure** **6**A). RNA pull‐down assay validated EIF4A3's binding capability to the flanking sequences of circFADS1, showing that only the downstream rather than the upstream flanking sequence of circFADS1 could be bound by EIF4A3 (Figure 6B; Figure S5A, Supporting Information). To verify EIF4A3 binding at the predicted sites, RIP assay was conveyed, which revealed EIF4A3 could bind to the downstream sites of circFADS1 (sites b, c, and d) (Figure 6C). To verify EIF4A3's effect on circFADS1, we silenced EIF4A3 in both HCC cells and overexpressed EIF4A3 in HepG2 cells. Then, we confirmed the knockdown efficiency (Figure S5B–E, Supporting Information). qRT‐PCR results showed that EIF4A3 knockdown significantly reduced circFADS1 expression (Figure S5B,C, Supporting Information). qRT‐PCR on 50 clinical HCC samples revealed a direct association between circFADS1 and EIF4A3 expression. (Figure 6D). However, qRT‐PCR and western blotting showed that EIF4A3 expression levels were not regulated by circFADS1 (Figure S5F,G, Supporting Information).

To further explore the relationship between EIF4A3 and circFADS1 in HCC cells, we performed western blot to investigate the protein expression levels of GSK3β, β‐catenin, and c‐MYC under conditions of altered circFADS1 and EIF4A3 expression. The results showed that in HepG2 cells, overexpression of EIF4A3 reduced the expression of GSK3β and increased the levels of β‐catenin and c‐MYC, while knockdown of circFADS1 reversed this effect (Figure 6E). In Hep3B cells, knockdown of EIF4A3 increased the expression of GSK3β and decreased the levels of β‐catenin and c‐MYC, while overexpression of circFADS1 counteracted this influence (Figure S5H, Supporting Information). Therefore, we designed corresponding rescue experiments, including EdU, colony formation, CCK‐8 assays, and apoptosis detection. The results showed that overexpression of EIF4A3 promoted HepG2 cell proliferation and inhibited apoptosis, effects that could be reversed by knockdown of circFADS1 (Figure 6F–I). Conversely, the knockdown of EIF4A3 had the opposite effect, which was counteracted by overexpression of circFADS1 (Figure S5I–L, Supporting Information). In summary, through binding to the downstream sequence of circFADS1, the biogenesis of circFADS1 was facilitated by EIF4A3, and further confirmed the EIF4A3/circFADS1/GSK3β/β‐catenin axis contributing to HCC progression.

### 2.9 CircFADS1 Facilitated HCC Cell Growth in Vivo

**Figure 7:**
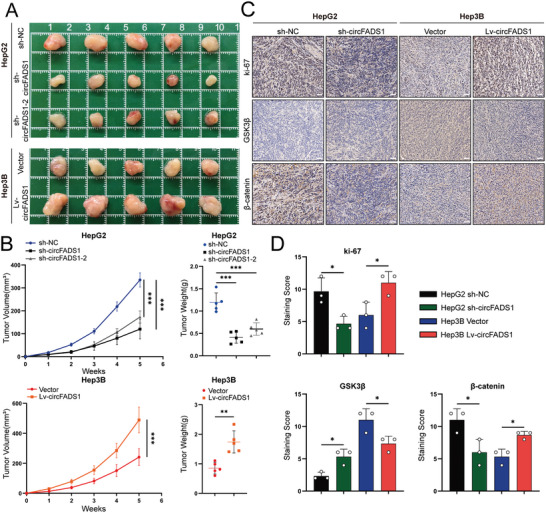
CircFADS1 accelerates the in vivo growth of HCC cells. A,B) Tumor size and weight of the subcutaneous tumor growth model injected with HCC cells (5 mice/group). C) IHC staining of the indicated proteins in the tumor samples obtained from the subcutaneous tumor growth model. Up, Ki‐67; middle, GSK3β; down, β‐catenin. D) IHC staining scores of xenograft tumors. ^*^ *p* < 0.05; ^**^ *p* < 0.01; ^***^ *p* < 0.001. Data were shown as mean ± SEM.

To examine circFADS1's effect on tumor development in vivo, HCC cells were subcutaneously injected into nude mice (**Figure** **7**A). Results showed subcutaneous tumors from HepG2 cells with circFADS1 knockdown were significantly smaller in either size or mass compared to control. In contrast, Hep3B cells with overexpressed circFADS1 exhibited a pronounced effect in promoting subcutaneous tumor growth (Figure 7B).

Xenografts from all four experimental groups were subjected to IHC staining with Ki67, β‐catenin, and GSK3β antibodies (Figure 7C). Analysis of the results revealed that Ki67 staining intensity decreased with circFADS1 knockdown, whereas circFADS1 overexpression enhanced Ki67 distribution. Simultaneously, IHC staining showed that β‐catenin expression decreased with circFADS1 knockdown and increased with circFADS1 overexpression, while GSK3β exhibited an opposite trend to β‐catenin (Figure 7D).

### 2.10 CircFADS1 Contributed to Lenvatinib Resistance

**Figure 8:**
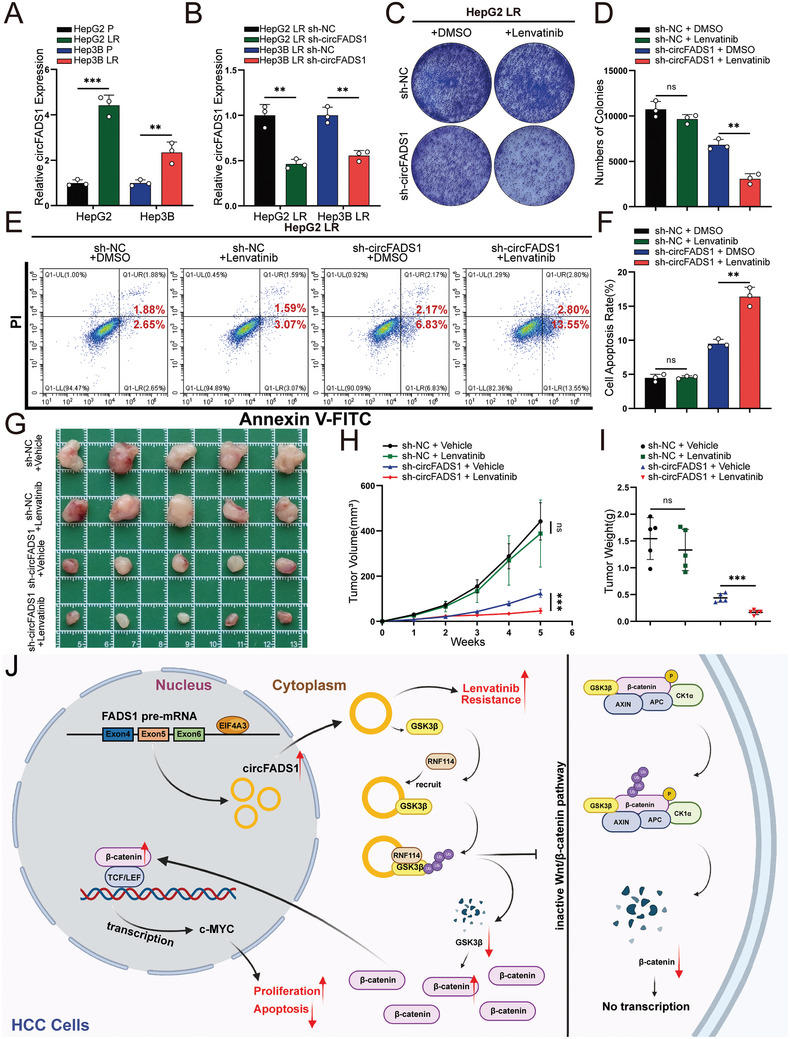
CircFADS1 is closely implicated in lenvatinib resistance. A) qRT‐PCR measured the expression levels of circFADS1 in lenvatinib‐resistant (LR) and parental (P) cell lines of HepG2 and Hep3B. B) qRT‐PCR confirmed the efficiency of circFADS1 knockdown in transfected HepG2 LR and Hep3B LR cell lines. C,D) Clonogenic assays were conducted on HepG2 LR cells with circFADS1 knockdown and their control group, following treatment with 10 µmol L^−1^ lenvatinib and an equivalent concentration of DMSO. E,F). Apoptosis cells were assessed on HepG2 LR cells with circFADS1 knockdown and their control group, following treatment with 10 µmol L^−1^ lenvatinib and an equivalent concentration of DMSO. G–I) Tumor size and weight of the subcutaneous tumor growth model injected with HepG2 LR cells and a daily dose of lenvatinib or control vehicle (5 mice per group). J) Schematic representation of the mechanism of circFADS1 in HCC cells (Created with BioRender.com). ^*^ *p* < 0.05; ^**^ *p* < 0.01; ^***^ *p* < 0.001. Data were shown as mean ± SEM.

To investigate whether circFADS1 plays a role in lenvatinib resistance, lenvatinib‐resistant (LR) cell lines were established, designated as HepG2 LR and Hep3B LR, with the parental cell lines called HepG2 P and Hep3B P. CCK‐8 assay was conducted to measure the IC50 of these cells treated with lenvatinib, and results revealed a markedly higher IC50 in LR cell lines compared to parental ones. (Figure S6A, Supporting Information). qRT‐PCR suggested that in either HepG2 cell lines or Hep3B cell lines, expression of circFADS1 was notably higher in LR cell lines (**Figure** **8**A). To investigate the impact of circFADS1 levels on lenvatinib resistance in HCC cells, we transfected HepG2 LR and Hep3B LR cell lines with knockdown plasmids targeting circFADS1 (Figure 8B). Initially, we treated LR cells with DMSO or 10 µmol L^−1^ lenvatinib for 24 h for subsequent investigation. CCK‐8, colony survival assay, and flow cytometry indicated that at a lenvatinib concentration of 10 µmol L^−1^, there was no significant impact on the viability, proliferation, or apoptosis of LR cells. However, inhibiting the expression of circFADS1 in LR cell lines significantly enhanced their sensitivity to lenvatinib (Figure 8C–F; Figure S6B–F, Supporting Information).

Next, we inoculated HepG2 LR cell lines with circFADS1 knockdown or control into the flanks of nude mice. From the time when the subcutaneous tumors became palpable (day 14), the mice were treated with 2 mg kg^−1^ lenvatinib or an equivalent amount of vehicle control daily (Figure S6G, Supporting Information). Similarly, in vivo, the tumor volume statistics showed the tumorigenic ability of the sh‐NC group of HepG2 LR cells was not significantly affected by lenvatinib, while the dose of lenvatinib significantly suppressed tumor growth in the sh‐circFADS1 group (Figure 8G,H). Furthermore, the tumor weights exhibited consistent characteristics (Figure 8I). In summary, circFADS1 is a crucial factor promoting lenvatinib resistance in HCC.

## 3 Discussion

Emerging evidence indicates that circular RNAs(circRNAs) are important contributing factors to tumors, holding promise to be a predictor and therapeutic target for cancers.^[^ ^29^ ^]^ In hepatocellular carcinoma (HCC), circRNAs also critically influence tumor onset and development.^[^ ^30^ ^]^

In this study, we identified a novel circular RNA, circFADS1, which exhibits elevated expression abundance in HCC. In HCC tissues, circFADS1 is expressed significantly higher than in adjacent non‐tumor tissues. Clinical HCC patients with higher levels of circFADS1 expression suffer notably larger tumor sizes, shorter survival times, poorer TNM staging, and worse Edmondson grades, implying circFADS1's potential as a predictive indicator for HCC. In experiments both in vitro and in vivo, circFADS1 actively drives HCC proliferation. Meanwhile, high expression of circFADS1 markedly inhibited the apoptosis of HCC cells. Interestingly, we found that EIF4A3 can enhance the biogenesis of circFADS1. Through high‐throughput RNA sequencing, Wnt/β‐catenin pathway was identified to be associated with circFADS1. Higher expression levels of circFADS1 lead to greater activation of the Wnt/β‐catenin pathway, with β‐catenin being a key protein in this process. It was found that GSK3β can be bound by circFADS1, ubiquitinated, and degraded by the specific E3 ubiquitin ligase RNF114, as identified by mass spectrometry. circFADS1 promotes the ubiquitination process by binding to both GSK3β and RNF114, ultimately inhibiting GSK3β‐mediated phosphorylation of β‐catenin and the subsequent degradation. Additionally, circFADS1 promotes resistance to lenvatinib in HCC. Thus, we found that EIF4A3‐mediated circFADS1 facilitates HCC progression via GSK3β/RNF114/β‐catenin axis and driving lenvatinib resistance.

The Wnt/β‐catenin pathway, crucial in development, homeostasis, and disease, is conserved across species. Also, abnormal Wnt/β‐catenin activation drives cancer initiation and progression.^[^ ^31^ ^]^ For the liver, research has shown that the homeostasis of the liver and the occurrence of HCC are closely associated with the Wnt pathway.^[^ ^32^ ^]^ Under normal circumstances, β‐catenin is sequestered in the cytoplasm, forming a complex with APC, AXIN1, and GSK3β, which phosphorylates its N‐terminus. This phosphorylation facilitates the ubiquitination of β‐catenin. Wnt activation recruits AXIN1 and GSK3β to the membrane, blocking the complex formation. This causes β‐catenin buildup in the cytoplasm, which then moves to the nucleus, binding to TCF and LEF to trigger transcription of targets like c‐MYC. Abnormal expression levels of GSK3β can also lead to similar outcomes.^[^ ^33^ ^]^ In this study, we applied high‐throughput transcriptome RNA sequencing and found that circFADS1 is closely related to the Wnt/β‐catenin pathway. Notably, the expression level of the key pathway factor β‐catenin is positively correlated with circFADS1, as well as the downstream genes. Additionally, the knockdown of circFADS1 can increase the expression of CCL4 and reduce the expression of PD‐L1. Meanwhile, functional experiments in vitro confirmed that circFADS1 exhibits oncogenic properties by maintaining high expression levels of β‐catenin and activating the Wnt/β‐catenin cascade.

Glycogen synthase kinase‐3 beta (GSK3β) belongs to the serine/threonine kinases regulating metabolism, cell cycle, and apoptosis. GSK3β is a critical regulator of the Wnt/β‐catenin signaling pathway. It phosphorylates β‐catenin, leading to its degradation. In many cancers, GSK3β inhibition leads to the β‐catenin buildup and translocation, promoting the transcription of oncogenes and tumor progression.^[^ ^34^ ^]^ After analyzing the results of the mass spectrometry of the RNA pulldown products, derived from the probes specific for circFADS1 and the control probe, an interesting finding emerged: circFADS1 has the potential to bind GSK3β. We confirmed the binding ability and dug out that GSK3β was downregulated by circFADS1‐meditated ubiquitination with specific E3 ubiquitin ligase RNF114. Ring finger protein 114 (RNF114) is a member of the RING‐type E3 ubiquitin ligase family, involved in various cellular processes through the ubiquitination and degradation of target proteins. RNF114 has been found to play a pivotal role in cancer, shaping metastasis, therapeutic response, and tumor development.^[^ ^35^, ^36^ ^]^ In this study, we found that circFADS1 can attach to RNF114 and act as a scaffold for RNF114 and GSK3β, promoting their interaction, and exhibiting oncogenic characteristics.

Lenvatinib, the most recent targeted therapy approved alongside sorafenib, is used for patients with advanced HCC and has been shown the ability of effectively prolonging overall survival (OS).^[^ ^37^ ^]^ Despite the effectiveness of lenvatinib in HCC, resistance to lenvatinib can develop, limiting its long‐term efficacy.^[^ ^38^ ^]^ Understanding the mechanisms of resistance is crucial for improving treatment outcomes and developing strategies to overcome it.^[^ ^39^, ^40^, ^41^ ^]^ Previous studies found that the Wnt/β‐catenin pathway was intimately linked with drug resistance in HCC.^[^ ^42^ ^]^ Lenvatinib‐resistant cell lines, which were established through long‐term drug induction and exhibited significantly higher expression levels than the parental cell lines, were used for the discovery of the role of circFADS1 in lenvatinib resistance. The resistant cell lines tolerated moderate lenvatinib levels and maintained growth. However, knocking down circFADS1 expression significantly increased the sensitivity of HCC to lenvatinib, indicating that suppressing the circFADS1 expression level can reverse lenvatinib resistance. Collectively, circFADS1 is a promising early marker and predictor for HCC, and a trackable indicator during lenvatinib therapy. Due to its role in the Wnt/β‐catenin pathway, it is also a potential target for immunotherapy. It holds promise as a new target for HCC therapy and for reversing lenvatinib resistance. Nevertheless, the mechanisms by which circFADS1 promotes lenvatinib resistance and modulates antitumor immunity remain to be further investigated.

## 4 Conclusion

Our data indicate that circFADS1, an upregulated circRNA in HCC, contributes to the progression of HCC both in vitro and in vivo. EIF4A3‐induced circFADS1 facilitates the binding of RNF114 to GSK3β and subsequent GSK3β ubiquitination, which activates the Wnt/β‐catenin pathway. Additionally, circFADS1 is a critical determinant in lenvatinib resistance. This study delves into the oncogenic mechanisms of circFADS1, offering novel targets for prognosis and therapy in HCC, with the potential to reverse lenvatinib resistance through circFADS1 modulation.

## 5 Experimental Section

### Clinical Samples

Following approval by the ethics committee (2023‐SRFA‐377), 50 matched pairs of HCC along with adjacent non‐tumor tissues were obtained during liver resection surgeries in the First Affiliated Hospital of Nanjing Medical University. All 50 HCC patients were undergoing surgery for the first time and had not received any prior antitumor treatment. Each patient provided written informed consent.

### RNA Extraction, Nucleocytoplasmic Separation, and qRT‐PCR

TRIzol from Invitrogen was utilized for RNA extraction from clinical tissues and HCC cells. The isolation of the nuclear and cytoplasmic fractions was conducted following the instructions of the PARIS kit (Invitrogen, USA). The reagents used for reverse transcription to synthesize the first strand of cDNA were obtained from Vazyme Biotech. The cDNA served as the template for qRT‐PCR. With the ABI 7900 detection system (Applied Biosystems, USA) and SYBR green from Vazyme Biotech, qRT‐PCR was conducted. Data were calculated using the 2−ΔΔCt method, with an internal reference gene of GAPDH. Table S1 (Supporting Information) contains the primer sequences used in all qRT‐PCR experiments.

### Cell Culture

Sourced from the Cell Bank of the Chinese Academy of Sciences (CASCB, China), serval cell lines of human HCC were utilized for experiments. The two‐step collagenase perfusion procedure proposed by Vondran, F.W. et al. was used in this experiment to isolate primary cells from patients undergoing hepatectomy.^[^ ^43^ ^]^ DMEM supplemented with 10% fetal bovine serum (all from Gibco, USA) served as the cell culture medium, and cells were incubated under standard conditions at 37 °C with 5% CO2.

### RNase R and Actinomycin D Treatment

RNase R (Lucigen, USA), a nuclease capable of specifically digesting linear RNA, was used to treat RNA extracted from HepG2 and Hep3B cells with the concentration of 2 U µg^−1^ at 37 °C for 10 min, with the control group receiving an equivalent volume of DMSO. qRT‐PCR was subsequently conducted. In addition, HCC cells (1 × 10^5^) were seeded into six‐well plates and grown overnight before being treated with 2 µg mL^−1^ actinomycin D (Sigma, USA). Cells were harvested at 6, 12, 18, and 24 h for RNA extraction, following qRT‐PCR.

### RNA FISH

To investigate the subcellular distribution of circFADS1 in HCC cells and tissues, Servicebio (Wuhan, China) synthesized Cy3‐labeled probes specifically designed for circFADS1, with the Olympus microscope (Tokyo, Japan) to perform the analysis. The probe sequence was: 5′‐GCCAGCCAGCCTGGGCTTAGGAAGAAGTATTTG‐3′.

### Plasmid Construction, siRNA Interference and Lentiviral Infection

Three different shRNAs targeting the circFADS1 junction site and random sequence were designed to create the circFADS1 knockdown and control groups(sh‐circFADS1, sh‐circFADS1‐2, sh‐circFADS1‐3, and sh‐NC), while lentiviruses carrying either the circFADS1 vector or an empty vector (Lv‐circFADS1/Vector) were used to establish the overexpression group; all constructs were sourced from GenePharma Co., Ltd. (Shanghai, China). Furthermore, RiboBio Biological Technology (Guangzhou, China) designed specific short interfering RNAs (siRNAs) targeting EIF4A3, β‐catenin, GSK3β, and RNF114, along with their corresponding negative controls (si‐NCs). Corues Bio (Nanjing, China) synthesized the plasmids for β‐catenin, GSK3β, and RNF114. Employed as the transfection agent, Lipofectamine 3000 (Invitrogen, USA) facilitated the introduction of lentiviruses, plasmids, and siRNAs. Detailed sequence information can be found in Table S1 (Supporting Information).

### CCK‑8 Assay

After ≈1000 HCC cells were plated into 96‐well plates, Cell replication capacity was assessed using the CCK‐8 assay kit (Dojindo, Japan). The cells were divided into five groups, with one group selected each day to be treated with 10 µL of CCK‐8 solution and incubated for 4 h for detection, while other cells kept incubation. On the following day, a new group of cells was selected for the CCK‐8 assay, and this process was repeated for five consecutive days. With the enzyme immunoassay analyzer (Thermo Fisher Scientific, Waltham, MA, USA), the viability of HCC cells was determined according to the absorbance at 450 nm.

### EdU Assay

EdU assays were performed using an EdU Kit from Beyotime to evaluate the proliferative capacity of HCC cells. Once the cells adhered and grew normally in 24‐well plates, EdU solution was added to the medium for incubation, followed by fixation with 4% paraformaldehyde and permeabilization with 0.3% Triton X‐100 (Servicebio, Wuhan, China). Subsequently, cells were treated with Click Reaction Buffer according to the instructions and stained with Hoechst 33342. Observation and imaging were performed with an Olympus microscope (Tokyo, Japan), with HCC proliferation was indicated by the proportion of cells stained with EdU among the total cell population.

### Clone Formation Assay

Around 600 HCC cells were seeded in each well of 6‐well plates, while HepG2 LR and Hep3B LR cells were seeded at 1 × 10^5^ cells per 6 cm dishes. All cells were cultured in DMEM for 14 days, with media changes every three days. Following a PBS wash, cells were fixed in 4% paraformaldehyde (Servicebio, Wuhan, China), stained with 0.1% crystal violet (Beyotime, China), and subsequently harvested.

### Apoptosis Analysis

Apoptotic cell levels were assessed with a FITC Annexin V Apoptosis Detection Kit (Vazyme, China). According to the protocol, ≈5 × 105. After performing two PBS washes, 100 µL of Binding Buffer was added to the cells, followed by 10 min staining of 5 µL of Annexin V‐phycoerythrin and 5 µL of propidium iodide, with 400 µL of Binding Buffer being added again. FACSCalibur Flow Cytometer (BD Biosciences, San Jose, CA, USA) was utilized to conduct the analysis of the proportion of apoptotic cells.

### Western Blot

Total cellular proteins were extracted using RIPA buffer obtained from New Cell & Molecular Biotech. Next, the extracted proteins were subjected to SDS‐PAGE for electrophoresis, followed by transfer onto PVDF membranes for further analysis. The blocking was conducted using Quick Blocking Buffer from Beyotime. After being left to incubate with the corresponding antibody overnight, TBST was utilized for washing. Subsequently, the PVDF membrane was incubated with secondary antibodies at room temperature for 2 h, followed by a TBST wash. An enhanced chemiluminescence (ECL) detection system was utilized to acquire the outcomes of western blot. All antibodies employed can be found in Table S2 (Supporting Information).

### RNA Seq

sh‐circFADS1 transfected cells and those with a negative control underwent RNA‐seq analysis. Transcriptome sequencing was performed by Bioprofile (Shanghai Bioprofile Co., Ltd.) using the Illumina HiSeq 2500 platform, including subsequent data processing (Table S3, Supporting Information).

### RNA Pull‑Down Assay and Mass Spectrometry

This study utilized a specific biotinylated circFADS1 probe from RiboBio (Guangzhou, China), along with a negative control probe. After harvesting and lysing 1 × 10^7^ HepG2 cells, they were incubated overnight with probe‐coated beads, prepared by 2 h of incubating 50 µL of beads with circFADS1 probe. Following elution, RNA‐protein complexes were isolated and subsequently analyzed through western blotting and mass spectrometry. After placing the aforementioned product into SDS‐PAGE for electrophoresis, the Fast Silver Stain Kit (Beyotime, China) was utilized for silver staining. A Q Exactive mass spectrometer (Thermo Fisher Scientific, CA, USA) was used to analyze the protein bands specific to the circFADS1 probe group in contrast to the control group. The results of MS are listed in Table S4 (Supporting Information).

### RNA Immunoprecipitation (RIP) Assay

Following the guidelines of RIP KIT from Millipore, the RIP assay for GSK3β and RNF114 was conducted. HCC cells were incubated with magnetic beads conjugated with either specific antibodies or anti‐IgG. The RNA obtained was then reverse‐transcribed into cDNA and analyzed by qRT‐PCR.

### Immunofluorescence (IF) Staining

After fixation and permeabilization, HCC cells were blocked with 10% serum for 30 min, followed by incubation with secondary antibodies at room temperature for 2 h. The samples were subsequently sealed and observed with an Olympus microscope (Tokyo, Japan).

### Cycloheximide (CHX) Treatment

HCC cells were cultured with 25 µg mL^−1^ cycloheximide (Selleck, China), which was an inhibitor of protein synthesis, and harvested at a certain time for protein extraction and Western blotting.

### In Vivo Nude Mouse Model

For studies on tumor growth in vivo, with the approval of the Institutional Animal Care and Use Committee of Nanjing Medical University (IACUC‐2206018), male nude mice aged 4 weeks were obtained from the Animal Core Facility at Nanjing Medical University (Nanjing, China). Nine distinct groups were randomly established, with five individuals per group. The mice were kept in a pathogen‐free setting under a standard light‐dark cycle.

Five groups were designated for subcutaneous injection of transfected HepG2 cells (sh‐NC, sh‐circFADS1, and sh‐circFADS1‐2) and Hep3B cells (Vector and Lv‐circFADS1), and the others were for HepG2 LR sh‐circFADS1 and HepG2 LR sh‐NC, to establish xenograft models. Approximately 2 × 10^6^ HCC cells in 10 mL of PBS were injected into the dorsal flank of the nude mice. During the growth for five weeks, tumor volume and mass were regularly recorded, and the mice were euthanized to collect subcutaneous tumor tissues.

### Immunohistochemical (IHC) Staining

Formalin‐fixed, paraffin‐embedded tissues were first deparaffinized in xylene and then rehydrated through a graded series of alcohols. Antigen retrieval was carried out in a pressure cooker for 15 min using a 10 mm sodium citrate buffer (pH 6.0). Subsequently, the samples were incubated with polyclonal antibodies against Ki67, β‐catenin, and GSK3β (all from Cell Signaling Technology) overnight, then exposed to secondary antibodies for 1 h.

Image capture was performed using a microscope from Leica Microsystems. The IHC staining scores were determined using the formula: IHC score = staining intensity (0: no staining; 1: light brown; 2: brown; 3: dark brown) × the proportion of positively stained cells (0: none; 1: < 25%; 2: 25–50%; 3: 50–75%; 4: > 75%). Two independent observers, blinded to the study conditions, assessed and scored all samples.

### Lenvatinib‑Resistant Cell Lines Establishment

Lenvatinib (E7080) was obtained from Selleck and was dissolved in DMSO as instructed. Briefly, cells were incrementally subjected to escalating concentrations of lenvatinib (ranging from 5 to 25 µmol L^−1^) to induce resistance. After a sour‐month induction period, two HCC cell lines resistant to lenvatinib, HepG2 LR and Hep3B LR, were successfully established.

### Bioinformatics Analysis

CircFADS1 sequence data were obtained from CircBase. Circinteractome (https://circinteractome.nia.nih.gov) was utilized to predict the RNA‐binding proteins (RBPs) associated with circFADS1. circRNADb (http://reprod.njmu.edu.cn/cgi‐bin/circrnadb/circRNADb.php) was employed to determine whether circFADS1 possesses open reading frames (ORFs) and the capacity to encode proteins.

### Statistical Analysis

Quantitative data are shown as means ± SD. Prism 8 software (GraphPad Software, La Jolla, CA, USA) was used to perform the data analysis. Student's *t*‐test compared two samples, while one‐way ANOVA analyzed multiple groups. All experiments were conducted in triplicate. Survival analysis used Kaplan–Meier for OS calculation and log‐rank test for comparison. Pearson's test (r, P) was used to assess the correlation between circFADS1 and EIF4A3 expression levels. statistical significance was indicated by ^*^ *p* < 0.05, ^**^ *p* < 0.01, and ^***^ *p* < 0.001.

## Conflict of Interest

The authors declare no conflict of interest.

## Supporting information



Supporting Information

Supporting Table 3

Supporting Table 4

## Data Availability

The data that support the findings of this study are available from the corresponding author upon reasonable request.
