# Supplementary material for: EIF4A3‐Mediated Biogenesis of CircFADS1 Promotes the Progression of Hepatocellular Carcinoma via Wnt/β‐Catenin Pathway
Source: Adv Sci (Weinh). 2025 Feb 18;12(14):2411869. doi: 10.1002/advs.202411869 (PMC11984884; doi:10.1002/advs.202411869)
Supplement: Supplementary file 1 — Supporting Information [file ADVS-12-2411869-s003.docx]

EIF4A3-mediated Biogenesis of CircFADS1 Promotes the Progression of Hepatocellular Carcinoma via Wnt/β-catenin Pathway

**Supplementary figures**

**
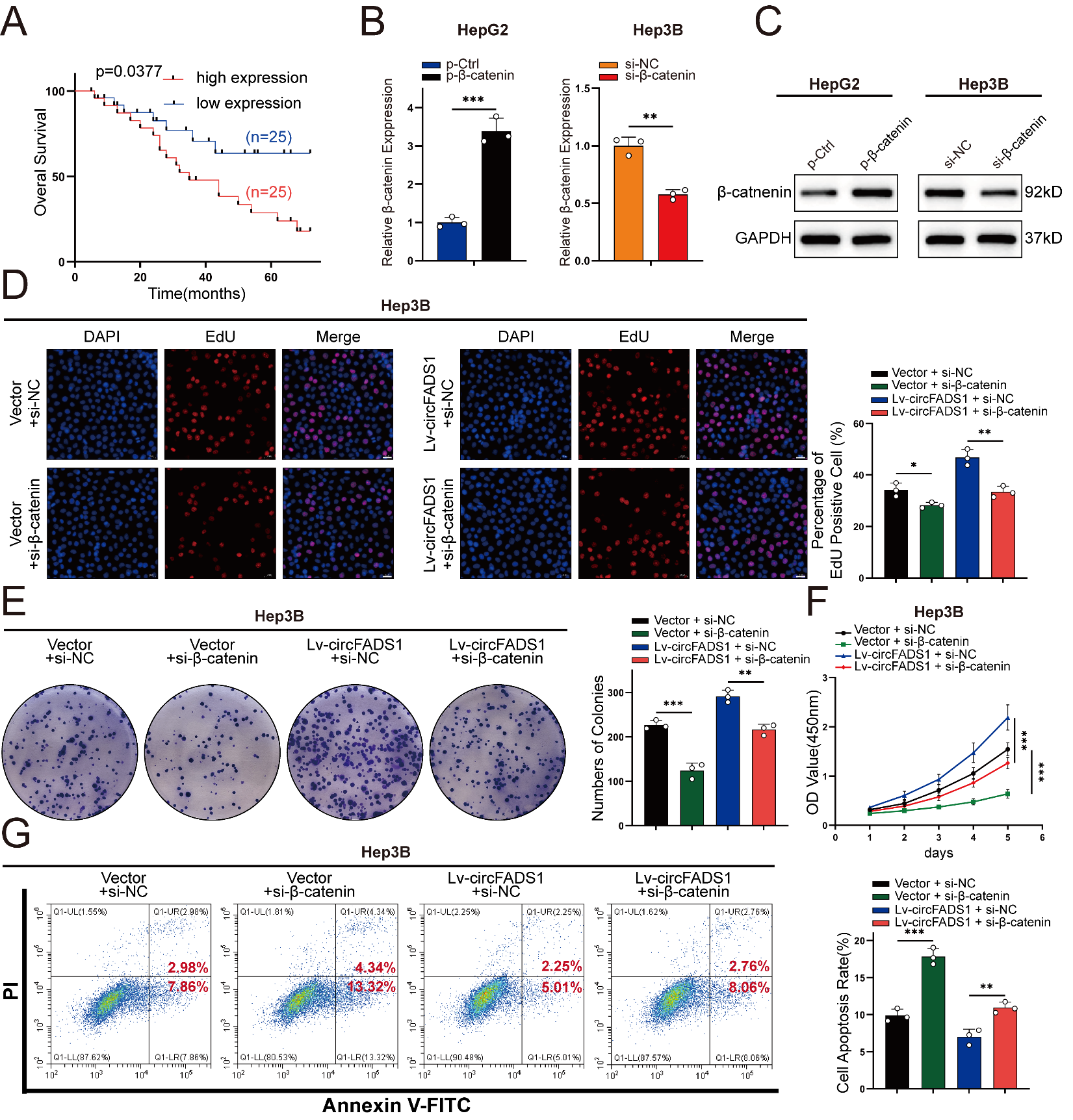
**

**Figure S1: CircFADS1 exerts its oncogenic effects through the Wnt/β-catenin pathway. A.** Kaplan-Meier plots of the overall survival of HCC patients with high (n = 25) and low (n = 25) levels of circFADS1. **B, C.** qRT-PCR and western blot confirmed the transfection efficiency of β-catenin overexpression and knockdown in HepG2 and Hep3B cells. Rescue experiments of overexpressing circFADS1 with knocking down β-catenin in Hep3B cells and their controls were conducted, including: **D.** EdU assays (Scale bar, 50 μm), **E.** colony formation assays, **F.** CCK-8 assays and **G.** apoptosis assessments. *p<0.05; **p<0.01; ***p<0.001. Data were shown as mean ± SEM.

**
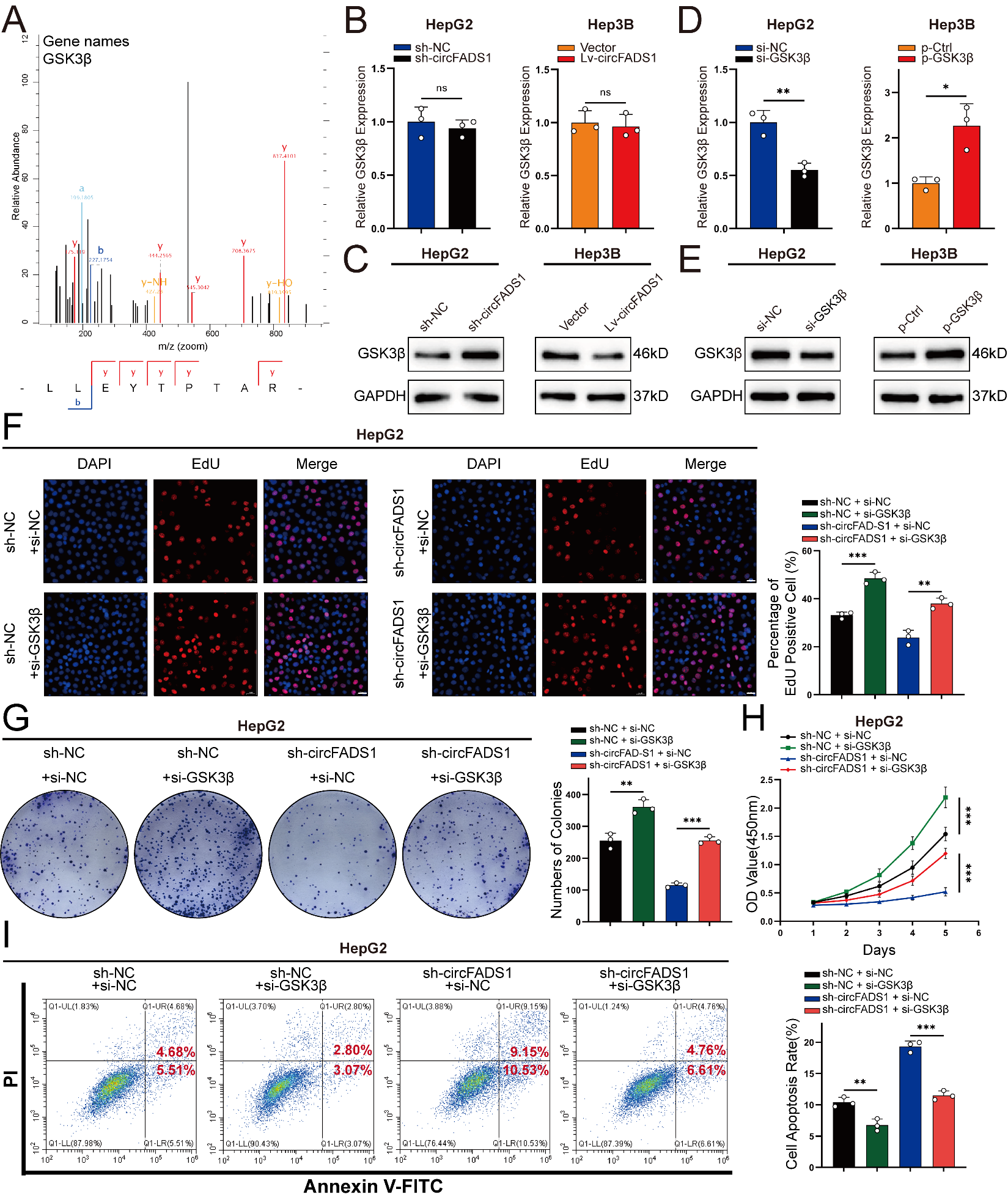
**

**Figure S2: Identification of GSK3β and the following rescue functional experiment. A.** Mass spectrogram of GSK3β protein. **B, C.** qRT-PCR and western blotting confirmed that circFADS1 regulates GSK3β at the post-transcriptional level rather than at the mRNA level. **D, E.** qRT-PCR and western blot confirmed the transfection efficiency of GSK3β knockdown and overexpression in HepG2 and Hep3B cells. Rescue experiments of knocking down circFADS1 with silencing GSK3β in HepG2 cells and their controls were conducted, including: **F.** EdU assays (Scale bar, 50 μm), **G.** colony formation assays, **H.** CCK-8 assays and **I.** apoptosis assessments. *p<0.05; **p<0.01; ***p<0.001. Data were shown as mean ± SEM.

**
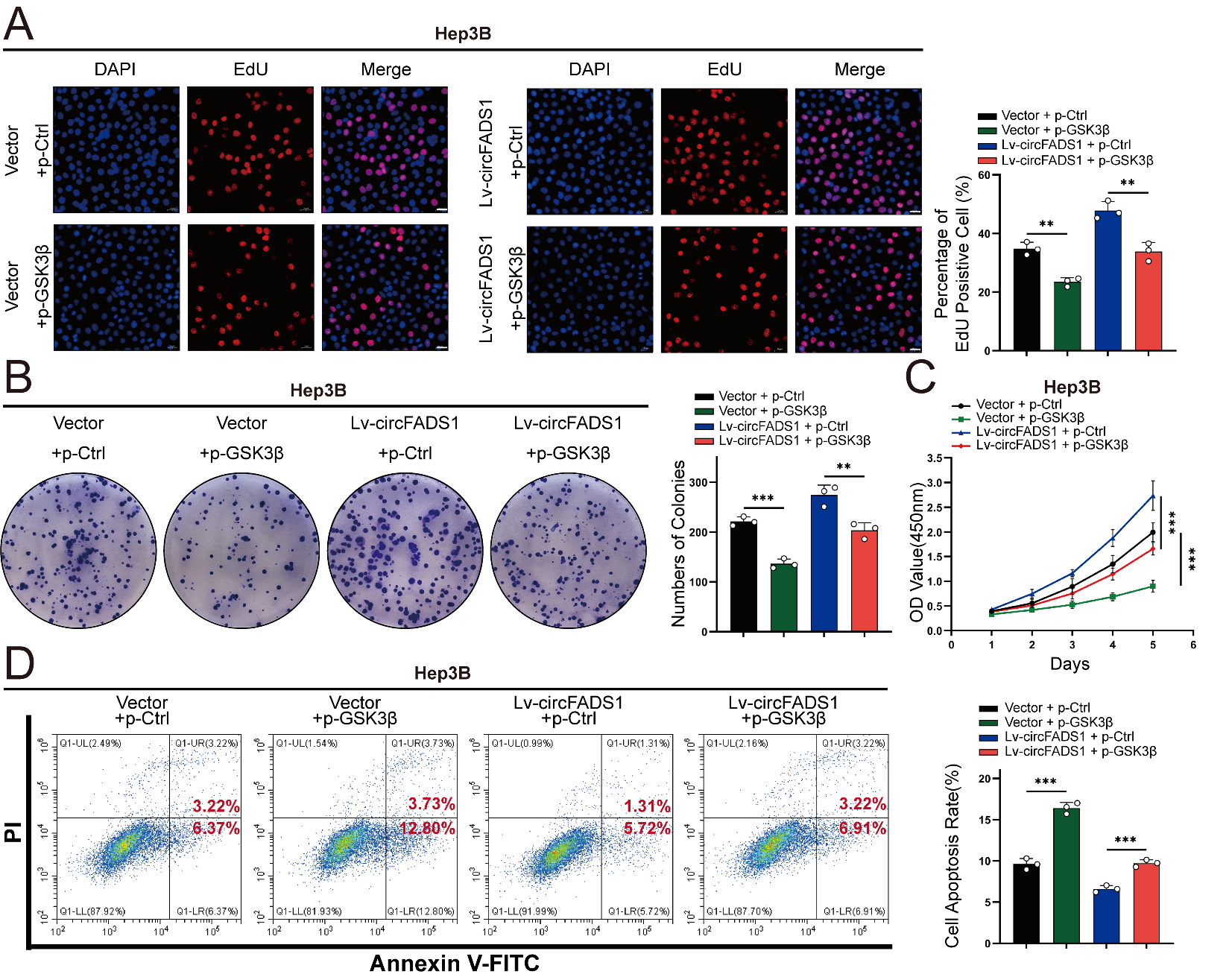
**

**Figure S3: GSK3β is the functional downstream mediator of circFADS1.** Rescue experiments of upregulation of circFADS1 with overexpressing GSK3β in Hep3B cells and their controls were conducted, including: **A.** EdU assays (Scale bar, 50 μm), **B.** colony formation assays, **C.** CCK-8 assays and **D.** apoptosis assessments. *p<0.05; **p<0.01; ***p<0.001. Data were shown as mean ± SEM.

**
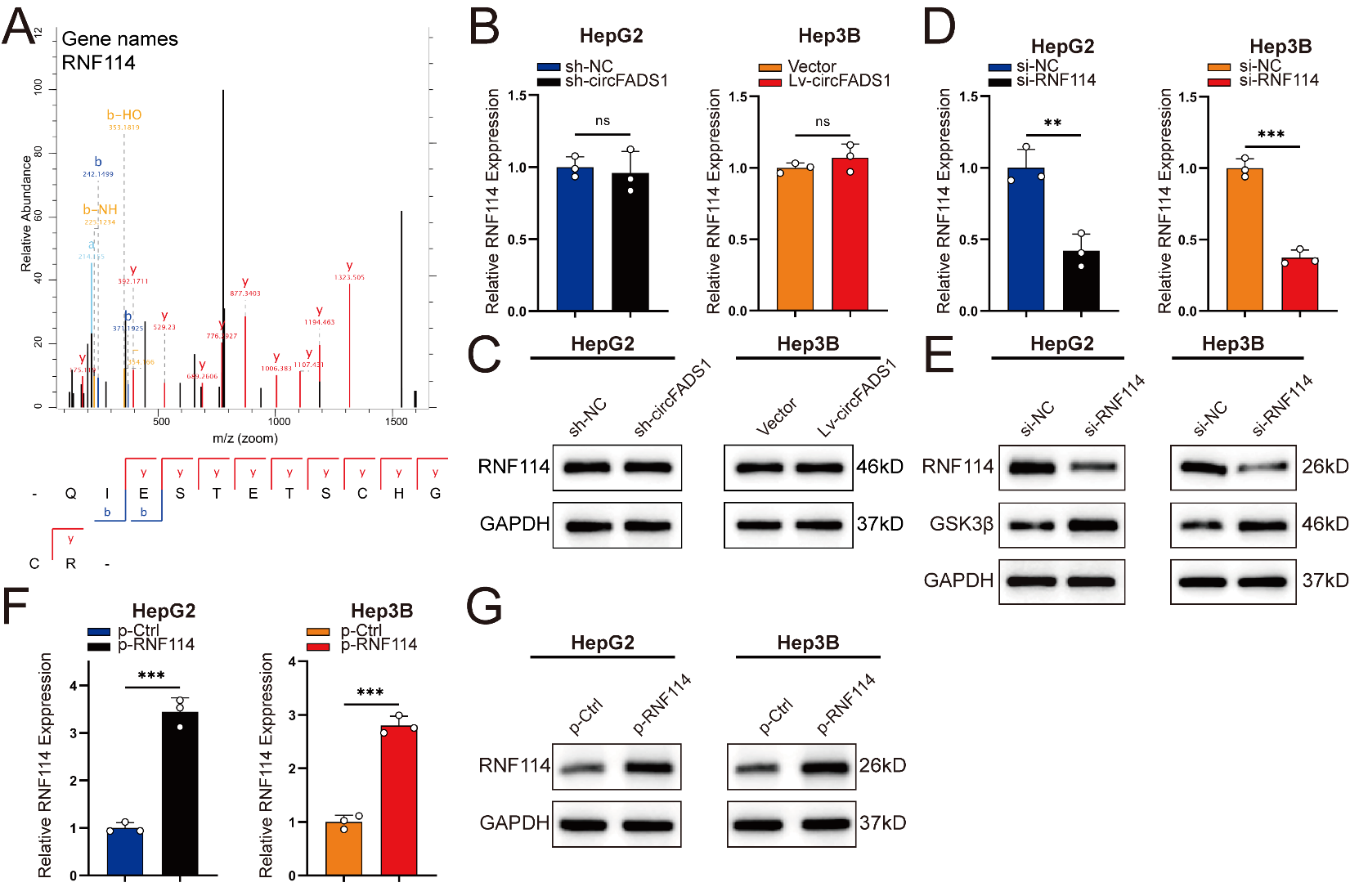
**

**Figure S4: Identification of RNF114 and RNF114-specific overexpression or knockdown. A.** Mass spectrogram of RNF114 protein. **B, C.** qRT-PCR and western blot were used to assess RNF114 expression levels during circFADS1 knockdown or overexpression. **D, E, F, G.** qRT-PCR and western blot confirmed the transfection efficiency of RNF114 knockdown and overexpression in HepG2 and Hep3B cells, and RNF114 knockdown results in the upregulation of GSK3β protein. *p<0.05; **p<0.01; ***p<0.001. Data were shown as mean ± SEM.

**
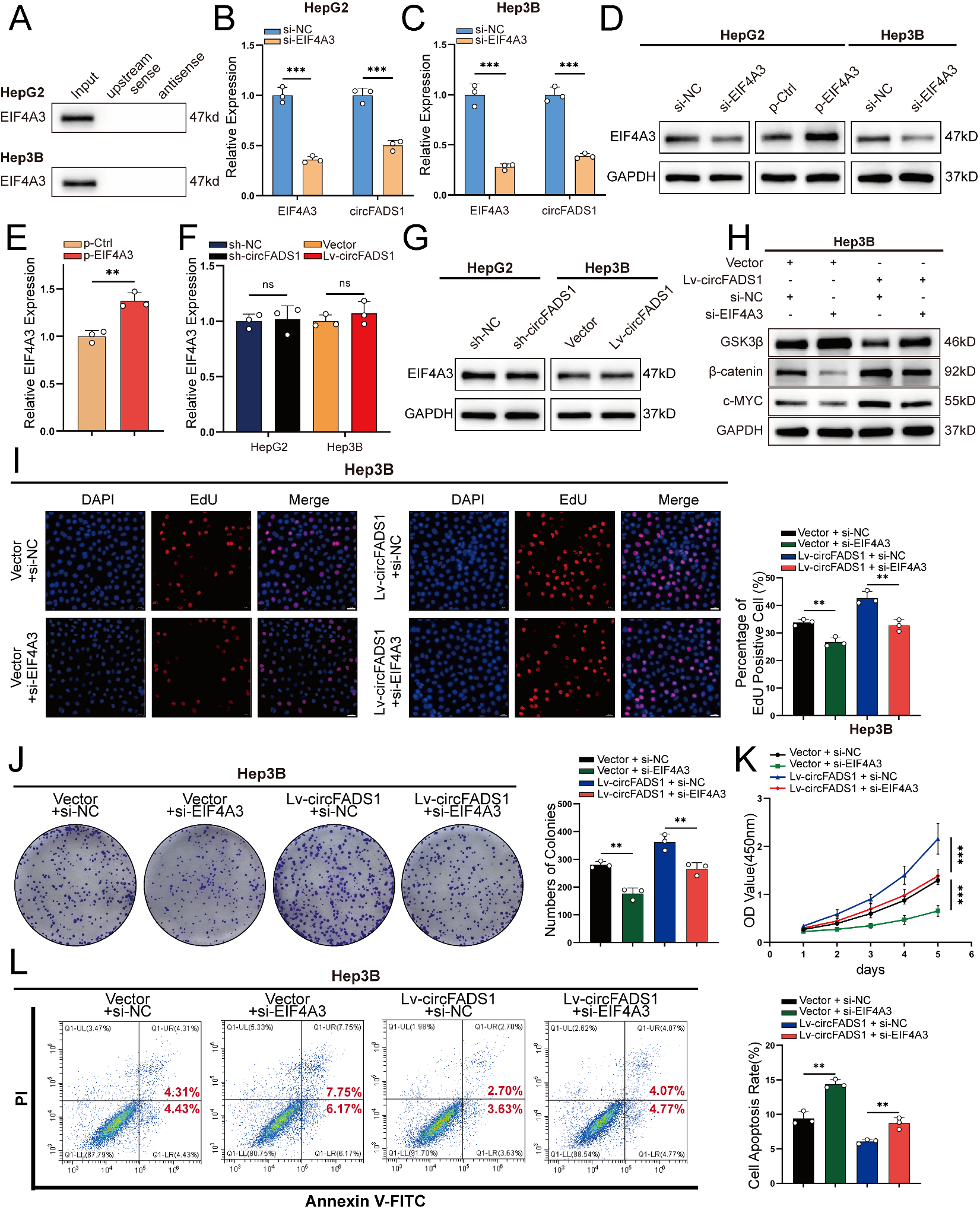
**

**Figure S5: A.** Pull-down assay with western blot verified the ability of EIF4A3 binding to the downstream of circFADS1 flanking instead of upstream. **B, C, D, E.** HepG2 and Hep3B cells were transfected with a siEIF4A3 or control. HepG2 cells were also separately transfected with p-EIF4A3 and its control. The transfection efficiency was evaluated by both qRT-PCR and western blot. Meanwhile, the expression of circFADS1 was also assessed by qRT-RCR. **F, G.** qRT-PCR and western blot analysis revealed that knockdown or overexpression of circFADS1 had no impact on EIF4A3 expression. **H.** Western blot of GSK3β, β-catenin and c-MYC under the condition of altered circFADS1 and EIF4A3 expression**.** Rescue experiments of knocking down EIF4A3 with overexpressing circFADS1 in Hep3B cells and their controls were conducted, including: **I.** EdU assays (Scale bar, 50 μm), **J.** colony formation assays, **K.** CCK-8 assays and **L.** apoptosis assessments.*p<0.05; **p<0.01; ***p<0.001. Data were shown as mean ± SEM.

**
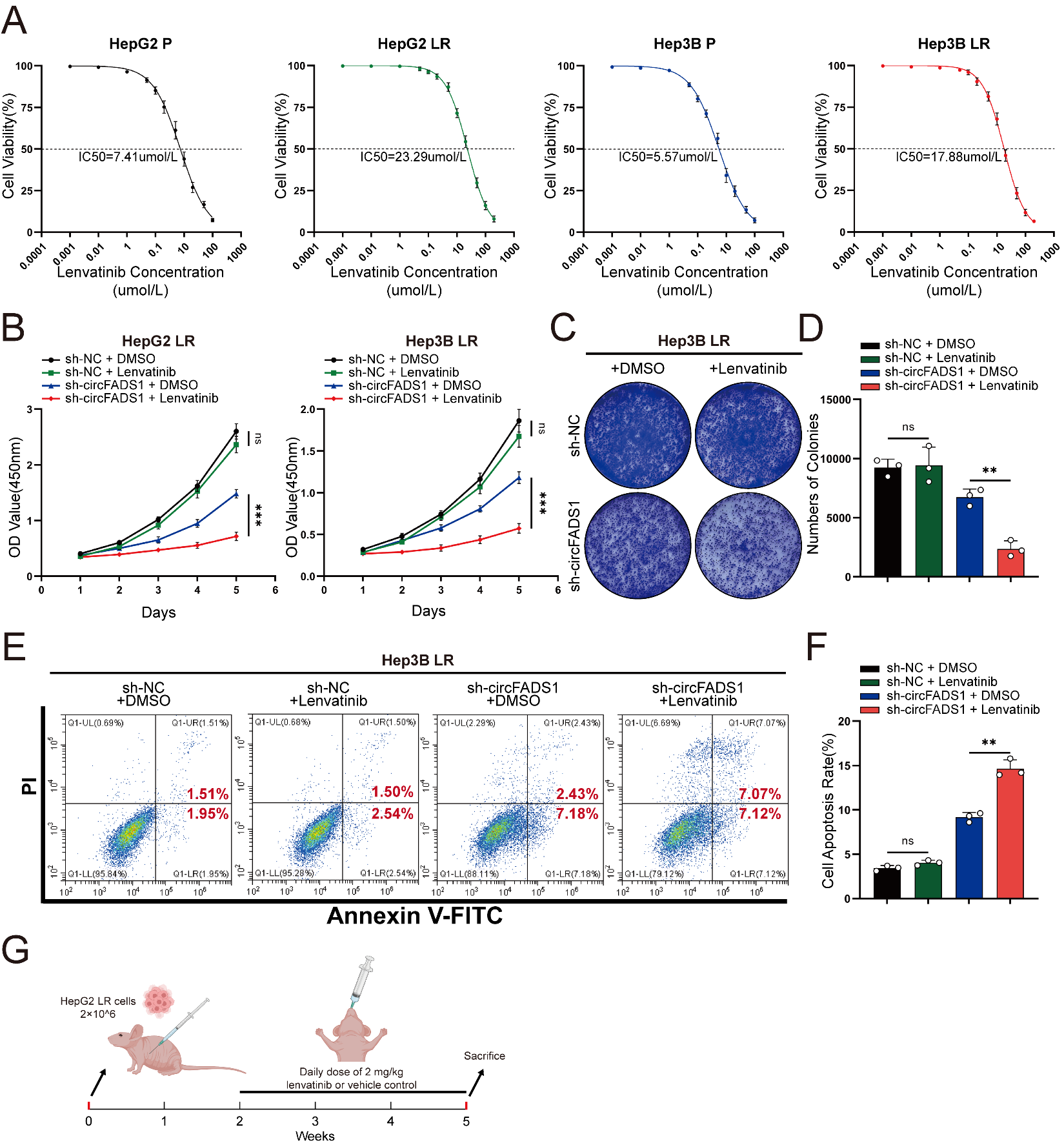
**

**Figure S6: Lenvatinib-resistant (LR) HCC cell lines were established to validate the role of circFADS1 in lenvatinib resistance. A.** Cell viability in HCC cells treated with varying concentrations of lenvatinib was assessed using the CCK8 assay, and relative IC50 values were calculated through nonlinear regression analysis with Prism 8 software. **B.** CCK-8 assays were conducted on HepG2 LR and Hep3B LR cells with circFADS1 knockdown and their control group, following treatment with 10 μmol/L lenvatinib and an equivalent concentration of DMSO. **C, D.** Clonogenic assays were conducted on HepG2 LR cells with circFADS1 knockdown and their control group, following treatment with 10 μmol/L lenvatinib and an equivalent concentration of DMSO. **E, F.** Apoptosis cells were assessed on HepG2 LR cells with circFADS1 knockdown and their control group, following treatment with 10 μmol/L lenvatinib and an equivalent concentration of DMSO. **G.** Schematic representation of the subcutaneous tumor model in nude mice injected with lenvatinib-resistant cells. *p<0.05; **p<0.01; ***p<0.001. Data were shown as mean ± SEM.

**Supplementary tables**

| **Table S1: Sequences of primers for qRT-PCR, probe for RNA pulldown and siRNAs used in this study** | | |
| --- | --- | --- |
| **Primer/RNA pulldown probe/siRNA** | | **Sequence (5’-3’)** |
| GAPDH | Forward primer | GGAGCGAGATCCCTCCAAAAT |
|  | Reverse primer | GGCTGTTGTCATACTTCTCATGG |
| GAPDH (divergent) | Forward primer | GAAGACTGTGGATGGCCCCT |
|  | Reverse primer | CAAATGAGCCCCAGCCTTCT |
| circFADS1 (divergent) | Forward primer | CAAATACTTCTTCCTAAGC |
|  | Reverse primer | CCCTTCAGGTGGCCAATCA |
| circFADS1 (convergent) | Forward primer | GTTGGTGGAACCACATGCAC |
|  | Reverse primer | GCTGGTGGTTGTACGGCATA |
| U6 | Forward primer | CGCAAGGATGACACGCAA |
|  | Reverse primer | GTGCAGGGTCCGAGGT |
| FASD1 | Forward primer | GTGGCTAGTGATCGACCGTAA |
|  | Reverse primer | ATTCTTGGTGGGCTCAAAGC |
| β-catenin | Forward primer | CAGCAGCAATTTGTGGAGGG |
|  | Reverse primer | GCAGCTGCACAAACAATGGA |
| GSK3β | Forward primer | TGGCAGCAAGGTAACCACAG |
|  | Reverse primer | CGGTTCTTAAATCGCTTGTCCTG |
| RNF114 | Forward primer | GCTGGTGCAAGCTTGGAAAT |
|  | Reverse primer | AGCCCAGGCTCCAGACTTAC |
| EIF4A3 | Forward primer | CGCGGACTCTGACATATGGCGACCACGGCCACGATG |
|  | Reverse primer | TCCCGCAGGCCCATGGTGTCG |
| circFADS1 (RNA pulldown probe) | sense sequence | CCUGGGCUUAGGAAGAAGUAUUUGU |
|  | anti-sense sequence | ACAAAUACUUCUUCCUAAGCCCAGG |
| si-β-catenin | sense sequence | AGACCUUCCUCCGUCUCCGCC |
|  | anti-sense sequence | CGGAGACGGAGGAAGGUCUGA |
| si-GSK3β | sense sequence | UCUUCCUUUUGUCUUUAUGUU |
|  | anti-sense sequence | CAUAAAGACAAAAGGAAGAAA |
| si-RNF114 | sense sequence | AUUUGGUAUCCGUGCUAUGGA |
|  | anti-sense sequence | CAUAGCACGGAUACCAAAUCU |
| si-EIF4A3 | sense sequence | AAUGAAAAAGACUUAGAACAA |
|  | anti-sense sequence | GUUCUAAGUCUUUUUCAUUAA |

**Table S2: List of antibodies**

| **Antibody** | **Catalogue NO.** | **Company** |
| --- | --- | --- |
| Rabbit Anti-β-catenin | 8480T | Cell Signaling Technology |
| Rabbit Anti-GADPH | 5174T | Cell Signaling Technology |
| Rabbit Anti-GSK3β | 12456S | Cell Signaling Technology |
| Rabbit Anti-PD-L1 | 13684T | Cell Signaling Technology |
| Rabbit anti-Ki67 | 9129T | Cell Signaling Technology |
| Anti-rabbit IgG, HRP-linked Antibody | 7074S | Cell Signaling Technology |
| Rabbit Anti-RNF114 | 14338-1-AP | Proteintech |
| Rabbit Anti-EIF4A3 | 17504-1-AP | Proteintech |
| Rabbit anti-ubiquitin | 10201-2-AP | Proteintech |
| Rabbit Anti-c-MYC | 10828-1-AP | Proteintech |
| Anti-IgG | ab172730 | Abcam |
| Anti-CCL4 | ab45690 | Abcam |
| Alexa Fluor 594-conjugated goat anti-Rabbit IgG | A-11012 | Invitrogen |
| Alexa Fluor 488-conjugated goat anti-Rabbit IgG | A-11008 | Invitrogen |
